# Successful extravascular implantable cardioverter-defibrillator implantation in a patient with recurrent transvenous implantable cardioverter-defibrillator erosion

**DOI:** 10.1016/j.hroo.2024.02.007

**Published:** 2024-03-10

**Authors:** Andrea Robinson, Sreedhar Billakanty, Eugene Fu, Anish Amin

**Affiliations:** OhioHealth Heart and Vascular Physicians, Section of Cardiac Electrophysiology, Department of Cardiology, Riverside Methodist Hospital, Columbus, Ohio

**Keywords:** Extravascular defibrillator, Transvenous ICD erosion, Device extraction, Device infection, Case report

## Introduction

The transvenous implantable cardioverter-defibrillator (ICD) has been demonstrated to reduce mortality in patients at risk for ventricular arrhythmias.^1^^,^^2^ Complications of transvenous ICD implantation may include direct cardiac injury, vascular injury or occlusion, and mechanical failure of the leads themselves.^3^ Infectious compromise of transvenous ICDs is an additional significant short- and long-term complication often necessitating system removal and adding to mortality and morbidity of affected patients. Routine use of antibiotic prophylaxis and antibiotic-impregnated envelopes can reduce the incidence of surgical site infections; however, cases of recalcitrant infection require novel solutions for management. We present a case of recurrent transvenous ICD pocket erosion successfully managed with an extravascular ICD (EVICD).

## Case report

A 63-year-old patient with a history of prior venous thromboembolism and a nonischemic cardiomyopathy was referred for implantation of a primary prevention ICD. The patient was diagnosed 6 months prior to his referral with acute hospitalization, at which time serial imaging demonstrated a left ventricular ejection fraction of 20% to 25%. Based on his presentation and the presence of complex ventricular ectopy on telemetry, a wearable cardioverter-defibrillator was recommended. Guideline-directed medical therapy was initiated with no subsequent improvement in left ventricular ejection fraction and symptoms consistent with New York Heart Association functional class II.

At the time of the initial implantation, the patient’s body mass index (BMI) was 18.2 kg/m^2^. Both subcutaneous and transvenous ICD implantation options were reviewed, and based on the presence of nonsustained ventricular tachycardia, a recommendation and decision were made for a single-chamber transvenous ICD. Routine antibiotic prophylaxis was administered prior to implantation. The device was then placed in the left pectoral region and axillary vein vascular access. A TYRX antibiotic pouch (Medtronic, Minneapolis, MN) was utilized and the incision was closed in 3 layers. Routine incisional care instructions were provided and an incision check was completed at day 8.

Seven months following implantation, the patient presented to device clinic after noting a small abrasion had dislodged at the mid portion of the incision. The device was readily visible and the patient was admitted electively for device removal with plans for reimplantation in the right pectoral space. There was no purulent drainage, erythema, or induration of the incision. The system was removed in its entirety with direct traction and the incision was closed primarily. Blood cultures were obtained and prophylactic intravenous antibiotics administered for 48 hours.

The second implantation was completed 48 hours later after cultures remained negative and using routine antibiotic prophylaxis and implantation of a dual-chamber ICD. The atrial lead was added for sporadic sinus bradycardia noted during the hospitalization for previous device explantation. The device was placed on the contralateral prepectoral space with axillary vein access. A TYRX antibiotic pouch and a CanGaroo pouch (Elutia, Silver Spring, MD) were utilized and the incision was closed in 3 layers. Standard care instructions were provided.

The patient presented 9 days following implantation to the hospital with purulent drainage from the incision site. A suture was noted to be protruding from the lateral edge of the incision and was removed. The site was reapproximated with steristrips and doxycycline was prescribed. Eight days following the hospitalization, the patient was seen in outpatient follow-up with recommendations to stop antibiotic therapy and return in 1 week for repeat evaluation of the incision. No erythema, induration, or drainage was noted during that visit. Twenty-five days following implantation, the patient presented with visible hardware through an incisional dehiscence. The device was removed without immediate complication.

**Figure 1:**
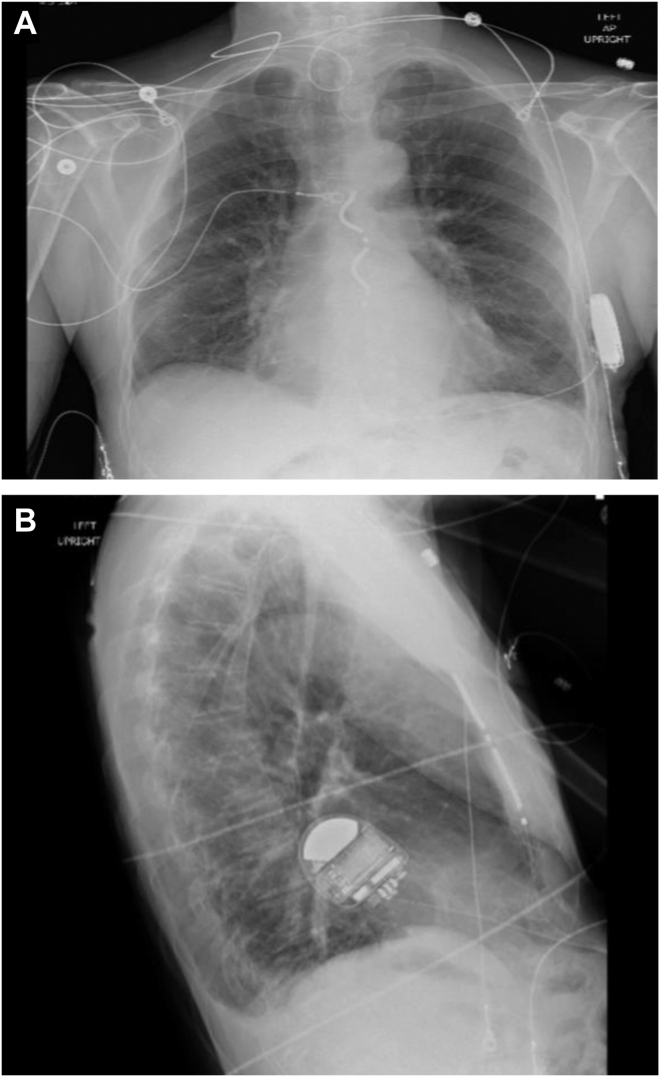
Follow-up chest radiograph following extravascular implantable cardioverter-defibrillator implantation demonstrating device positioning. **A:** Anterior view chest x ray. **B:** Lateral view chest x ray.

Following system extraction, the patient was referred for consideration of EVICD implantation as part of the global investigational device exemption study. Ultimately, the patient was deemed to be a suitable candidate for implantation. The patient underwent EVICD implantation 378 days following his original ICD implantation with no significant change in BMI. The ICD device and lead implantation consists of the creation of a subxiphoid pocket allows for tunneling of the ICD coil and a mid-axillary pocket allows for implantation of the generator. The ICD coil is placed in the substernal space using blunt dissection of the diaphragmatic attachments and advanced under lateral fluoroscopic visualization. The lead is tunneled to the mid-axillary pocket using a proprietary tunneling instrument. The device is recommended to be preferentially placed above the serratus anterior, but given the concern for erosion, an intramuscular position was elected (Figures 1A and 1B). The device was implanted per protocol and demonstrated implant R waves of 2.9 mV and underwent successful defibrillation using 15 J of energy. A postimplantation incision check at 8 days was satisfactory. The patient has subsequently been followed for over 18 months with no signs of infection or erosion and stable electrical parameters.

## Discussion

Cardiac implantable electronic devices (CIEDs) including pacemakers, ICDs, and cardiac resynchronization therapy devices are being implanted at increasing rates.^1^ Infectious complications related to CIEDs continue to remain a significant concern, leading to increased morbidity and mortality in affected patients.^2^^,^^3^ Prophylactic antibiotic administration and the use of antibiotic-impregnated envelopes can reduce the infection-related complications of CIEDs.^2^ Despite these strategies, selection of appropriate devices, and specifically the use of nontransvenous systems when available, should be considered.

Patient demographics which portend increased risk of system infection have been well described and include advanced heart failure, renal disease, and reduced BMI.^3^^,^^4^ This case highlights a patient with serial risk factors for system erosion. Subcutaneous ICDs have been described as a suitable alternative for patients with transvenous ICD infection.^5^ Owing to the subcutaneous position, lower rates of device complication related to pocket erosion and infection have been reported in both replacement and de novo procedures. Knops and colleagues^6^ reported 50% decrease in infectious complication with subcutaneous ICD implantation vs single-chamber transvenous implantation. Overall device-related complications were also lower in subcutaneous ICD cohort compared with the transvenous ICD cohort in that example.^6^ Real-world infection data remain significantly higher with subcutaneous ICD postapproval study results describing a 3.3% infection rate.^7^

In patients with low BMI, subcutaneous ICD implantation remains a challenge owing to the large generator size (60 cm^3^). Early observational implantation experience described a significant learning curve to reduce the incidence of infection as well as significantly wider rates of infection, some as high as 17%.^8^^,^^9^ The logistics of implantation and operator comfort in the mid-axillary and xyphoid space may play a role in managing surgical site infections and likely improve with experience. Appropriate positioning of a large generator, however, remains a challenge especially in patients with low BMI. The EVICD emerges as a new solution for patients at high risk for infection, with many of the advantages that an entirely subcutaneous device affords. In addition to the historical benefits, the EVICD offers a generator that is approximately half the volume of the subcutaneous ICD at 33 cm^3^.^10^ The EVICD PIVOTAL study included patients with 6 months of follow-up and reported an infection rate of 4.1%, with only 4 (1.3%) patients requiring system removal, and the remaining 9 were treated with antibiotic therapy.^10^ An updated dataset with 18-month follow-up included 2 additional reported infections and no reports of mediastinitis.^11^ Follow up of EVICD PIVOTAL study is ongoing and will continue to provide long-term safety data. Compared with subcutaneous ICD, EVICD can also deliver antitachycardia pacing therapy for ventricular arrhythmias, pause prevention pacing, and provide low-energy defibrillation. Hence, the EVICD may be an ideal implant choice in patients with multiple risk factors for infection and reduced BMI.

## Conclusion

Our case report highlights the potential of an EVICD system in patients who are at increased risk of transvenous system infection and erosions. Patients with low BMI should especially be considered for nontransvenous system implantation.
